# Influence of Overcrowding in the Emergency Department on Return Visit within 72 H

**DOI:** 10.3390/jcm9051406

**Published:** 2020-05-09

**Authors:** Dong-uk Kim, Yoo Seok Park, Joon Min Park, Nathan J. Brown, Kevin Chu, Ji Hwan Lee, Ji Hoon Kim, Min Joung Kim

**Affiliations:** 1 Department of Emergency Medicine, Yonsei University College of Medicine, 50 Yonsei-ro, Seodaemun-gu, Seoul 03722, Korea; KDU919@yuhs.ac (D.-u.K.); PYS0905@yuhs.ac (Y.S.P.); KEROKEROPI@yuhs.ac (J.H.L.); JICHOON81@yuhs.ac (J.H.K.); 2 Department of Emergency Medicine, Inje University Ilsan Paik Hospital, 170 Juhwa-ro, Ilsanseo-gu, Goyang-si, Gyeonggi-do 10380, Korea; aero7@outlook.kr; 3 Emergency and Trauma Centre, Royal Brisbane and Women’s Hospital, Butterfield Street, Herston QLD 4029, Australia; nathan.brown3@health.qld.gov.au (N.J.B.); Kevin.Chu@health.qld.gov.au (K.C.); 4 Faculty of Medicine, The University of Queensland, Brisbane QLD 4072, Australia

**Keywords:** emergency department, crowding, return visit, admission, patient satisfaction, quality of healthcare

## Abstract

This study was conducted to determine whether overcrowding in the emergency department (ED) affects the occurrence of a return visit (RV) within 72 h. The crowding indicator of index visit was the average number of total patients, patients under observation, and boarding patients during the first 1 and 4 h from ED arrival time and the last 1 h before ED departure. Logistic regression analysis was conducted to determine whether each indicator affects the occurrence of RV and post-RV admission. Of the 87,360 discharged patients, 3743 (4.3%) returned to the ED within 72 h. Of the crowding indicators pertaining to total patients, the last 1 h significantly affected decrease in RV (*p* = 0.0046). Boarding patients were found to increase RV occurrence during the first 1 h (*p* = 0.0146) and 4 h (*p* = 0.0326). Crowding indicators that increased the likelihood of admission post-RV were total number of patients during the first 1 h (*p* = 0.0166) and 4 h (*p* = 0.0335) and evaluating patients during the first 1 h (*p* = 0.0059). Overcrowding in the ED increased the incidence of RV and likelihood of post-RV admission. However, overcrowding at the time of ED departure was related to reduced RV.

## 1. Introduction

Return visit (RV) is often used as a quality indicator for the emergency department (ED), because the general idea is that RV is caused by premature discharges at the initial visit, missed diagnosis, or failure of treatment or discharge planning [1,2,3,4,5]. RV not only delays proper treatment of patients but also increases resource use and medical costs [6,7]. The causes of RV are very complex and multifactorial. Besides medical error, misdiagnosis, and delayed diagnosis as doctor-related factors, other factors such as disease progression, lack of improvement, or patient’s concern and fear about their condition contribute to RV [4,5,8,9,10]. Some studies have addressed the relationship between RV and the healthcare system, such as hospital bed shortages and ED overcrowding [11,12,13].

Worldwide, ED overcrowding is a healthcare problem, and has led to increased misdiagnosis and medication errors [14,15,16,17,18]. Emergency physicians must always provide timely first aid to emergent patients; therefore, when the ED is overcrowded, doctors hasten the discharge process of patients to prepare an empty bed for new emergent patients. Such an increase in premature discharge may sometimes lead to inadequate discharge, which can increase the rate of RV. To date, few studies have explored the relationship between ED overcrowding and RV, and no significant effect has been reported [8,19,20]. Verlest et al. reported that the number of patients occupying ED during the first 8 h of the index visit was unassociated with the occurrence of RV [19]. Hu et al. compared the occurrence of the RV with and without admission, and found that the number of whole-shift patients of the index visit did not differ between the two groups [8]. In a study by Hayward et al., ED occupancy level was not a significant factor for predicting admission in RV patients [20]. These results can differ on the basis of the definition of ED overcrowding. Therefore, a more sophisticated definition of the overcrowded state of index visits is needed to clarify the correlation between ED overcrowding and RV. ED overcrowding at the time of arrival of an index visit may affect RV, but overcrowding at the time of departure may be a more important factor. Moreover, in the ED, patients who are undergoing emergency evaluations and those awaiting a hospital bed after being advised hospitalization are together, and the impact of the number of patients under evaluation and the number of potential inpatients on the occurrence of RV may differ.

In this study, we specifically defined criteria for ED overcrowding, considering several time points of index visits and the composition of patients occupying the ED. The primary objective was to identify the effect of ED overcrowding at index visit on the occurrence of RV. The secondary objective was to determine whether ED overcrowding of index visit is a factor that causes RV with admission (RVA) compared with RV with no admission (RVNA). We hypothesized that overcrowded ED at the index visit would increase the occurrence of RV and would be a factor that induces RVA rather than RVNA.

## 2. Materials and Methods

### 2.1. Study Design and Setting

We conducted a retrospective observational study at the ED in a 2000-bed tertiary-care center, located within the capital of South Korea. The ED is divided into two areas as adult and pediatric ED; the adult ED covers patients older than 16 years. On average, 70,000 patients visit the adult ED annually and admission rate is approximately 24%. We included patients who were treated and discharged home in the adult ED between October 2017 and June 2019. We excluded patients who were transferred to other hospitals or discharged as hopeless patients after ED treatment. Furthermore, to analyze the first index visit, we designated visits within the period to define “short-term revisit” from the prior visit and these were excluded. All data were anonymized and were collected from the hospital information system, and this study was exempted from the obligation to obtain informed consent by the institutional review board committee of this hospital.

In our hospital, on patient arrival at the ED, the triage nurse classifies the acuity of patient using the Korean Triage and Acuity Scale (KTAS), a five-level classification system (1 = resuscitation, 2 = emergency, 3 = urgent, 4 = less-urgent, 5 = non-urgent). If the patient is unstable, such as with altered mental status or shock, the patient is directly sent to the monitoring area and treatment is initiated. Otherwise, the patient is examined by an emergency doctor in the doctor’s office and assigned to the monitoring area, bed area, chair area, or fast-track according to the patient’s condition and medical problems. The monitoring area has 15 beds, including two beds for resuscitation, and the bed area has 26 beds. There were 20 recliner chairs in the chair area, and the fast-track area has no specified seats. In each area, emergency doctors initially evaluate and treat patients, and they consult a specialist if the patient needs to be hospitalized or needs special care, including emergency procedures. Patients who are identified for hospitalization are taken to the hospital wards; however, in the absence of empty hospital beds, patients are admitted in the ED until a hospital bed becomes available. This ED does not operate any system to follow up the patients discharged from the ED; therefore, patients who need additional care after discharge are scheduled for outpatient clinic visits at the time of ED discharge.

### 2.2. Definition and Data Processing

We defined the RV as returning to the ED within a short-term period of 72 h from the ED departure time of the index visit. Despite a lack of supporting evidence, 72 h is the time period used in 63% of previously reported studies [21]. Furthermore, the RV was divided into the RVA and RVNA, according to the treatment outcome when the patient returned to the ED. Patients who died in the ED or were transferred to another hospital were included in the RVA.

**Figure 1:**
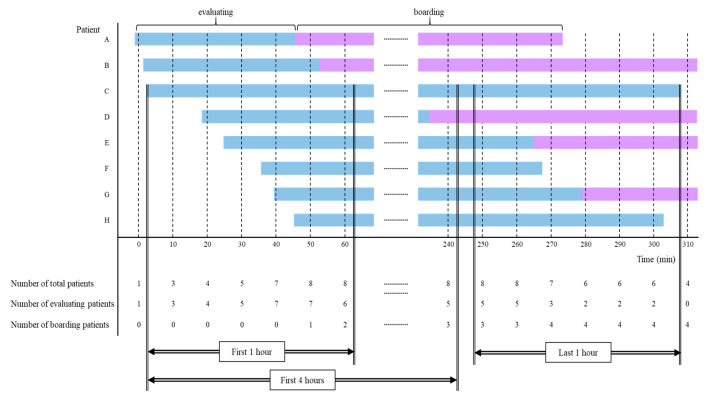
Data processing to reconstruct crowding indicators from an individual patient’s time-related factors. Bars represent the stay of individual patients in the emergency department; the blue bar represents an evaluating phase and the purple bar indicates the boarding phase. If the patient’s stay spanned the time of the 10 min interval, the patient was added to the number of patients at that time. For crowding indicators, the average number of patients during the first 1 h and 4 h and the last 1 h were calculated. For example, in Patient C, the average number of total patients during the first 1 h was (3 + 4 + 5 + 7 + 8 + 8)/6 = 5.8 and the average number of boarding patients during the last 1 h was (3 + 3 + 4 + 4 + 4 + 4)/6 = 3.7.

To define the overcrowding of the ED, we decided to consider the number of total patients occupying the ED, the number of evaluating patients to estimate the overload in the throughput of the ED, and the number of boarding patients to reflect the blocking of ED output. For this, we adapted three time components: ED arrival time, disposition (admission or discharge) decision time, and the ED departure time of all patients who visited the adult ED during the study period. From these time components of each patient, we summed up all relevant numbers and evaluating (from ED arrival to the disposition decision) and boarding (in the ED, awaiting a hospital bed after the decision to admit) patients by reconstructions set at 10 min intervals (Figure 1).

In this ED, the average time from the ED arrival to the decision of admission or discharge was 4 h. Therefore, we determined the level of ED crowding at the time of the index visit as the average number of occupying patients for 4 h from the time of ED arrival. Moreover, the first 1 h from ED arrival and the last 1 h before ED departure were designated as additional timeframes for calculating the crowding indicators. With regard to total patients, the average number of total patients during the first 4 h (TF4h), the first 1 h (TF1h), and the last 1 h (TL1h) were calculated. Similarly, three indicators were calculated for each evaluating and boarding patient; the average number of evaluating patients during the first 4 h (EF4h), the first 1 h (EF1h), and the last 1 h (EL1h); and the average number of boarding patients during the first 4 h (BF4h), the first 1 h (BF1h), and the last 1 h (BL1h). If the patient remained in the ED for less than the time span of crowding indicators (1 and 4 h), the average value during the patient’s stay was used for crowding indicators.

We assumed that the influence of overcrowding would not increase proportionally with the number of patients in the ED, and that the number of patients would not have a negative effect, to a certain extent. The number of patients would begin to affect overcrowding from a certain point; however, there are no previous studies that investigated this critical point. In some studies, ED occupancy rate was divided into four quartiles, and the quartile with the highest occupancy was defined as overcrowding that exceeded the treatment capacity of the ED [19,22]. We also designated the third quartiles of each of the total, evaluating, and boarding number of patients as the critical points, and defined the ED as overcrowded when the level of crowding indicators of the index visit exceeded these critical points.

### 2.3. Study Variables

All variables were retrieved from hospital information system and electronic medical records. We collected patient demographic data (sex and age), factors of ED visit (mode of transport (self or ambulance), direct ED visit, or transfer from another hospital), and factors of patient clinical severity such as urgency level rated by KTAS and the presence of severe medical conditions designated on the basis of the survival risk ratio by the central medical center under the Ministry of Health and Welfare. The following characteristics of the patient’s problem and ED treatment were also collected: the chief complaint; the reason of the ED visit (medical problem or not); whether the attending physician was involved for the patient care; assigned treatment area; whether specialty consultation was requested; ED arrival time and date; time from ED arrival to designated area (door to area time); length of stay (LOS) in the ED; and whether specific evaluation such as blood tests, computed tomography (CT), and magnetic resonance imaging (MRI) were conducted in the ED. For analysis, we categorized arrival time into four time zones (0–6, 6–12, 12–18, and 18–24) and ED visit date into binominal variables of weekdays and weekends, as well as into nominal variables about season (December to February as winter, March to May as spring, June to August as summer, and September to November as autumn). Crowding indicators of index visit were transformed into binominal variables (overcrowded or not) on the basis of the critical point (the third quartile value).

### 2.4. Statistical Analysis

We first compared the patient characteristics of index visits with and without RVs. The nominal variables were compared by chi-squared test and expressed in number and percentage. The continuous variables were analyzed by Mann–Whitney U test considering the skewness, and were presented as median and interquartile range (IQR). The univariable logistic regression was used to identify the factors affecting RV incidence, and the multivariable logistic regression was undertaken by adjusting confounding variables with *p*-value <0.1 as a result of univariable regression. Adjusted odds ratio (OR) with 95% confidence interval (CI) of each crowding indicator was presented. Second, the characteristics of RVA and RVNA were compared in order to investigate whether overcrowding at the index visit showed significant differences between RVA and RVNA. Moreover, we identified the effect of overcrowding on RVA through multivariable logistic regression by adjusting confounding variables. The analysis was conducted using SAS (version 9.4, SAS Inc, Cary, NC, USA), and determined to be statistically significant when the *p*-value was <0.05.

## 3. Results

**Figure 2:**
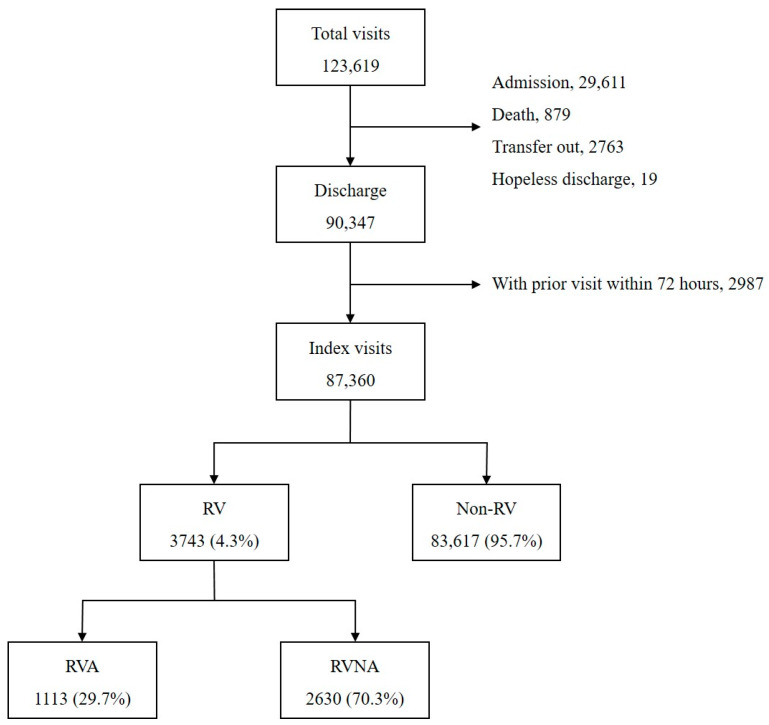
Study patient disposition. RV, return visit; RVA, return visit with admission; RVNA, return visit with no admission.

There were a total 123,619 ED visits during the study period (Figure 2). The ED treatment resulted in 29,611 (24.0%) admissions, 879 deaths, 1763 transfers to other hospitals, and 19 hopeless discharges, which were excluded from the analysis. Of the 90,347 discharges, 2987 cases with a history of prior ED care within 72 h were excluded. Finally, 87,360 index visits were included in the analysis; 4.3% (3743) of these index visits led to RV within 72 h, and 1113 (29.7% of RV) were admitted as a result of RV.

**Figure 3:**
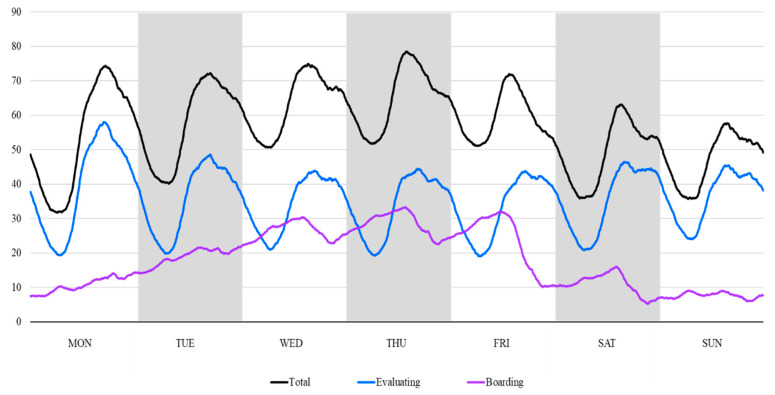
The hourly distribution by day of week of patients occupying the emergency department.

The count of measurements at 10 min intervals for total, evaluating, and boarding patients was 91,872. The hourly distribution by day of week of the number of occupying patients is shown in Figure 3. The number of the total and evaluating patients were lowest at dawn and highest in the afternoon, with similar patterns for each day of the week. The number of boarding patients gradually increased from Monday to Friday, and then decreased on Friday afternoon. The median (IQR) value was 56 (44–68) for the total number of patients, 35 (26–44) for the number of evaluating patients, and 14 (8–26) for the number of boarding patients. Therefore, the third quartile, the critical point of overcrowding, was 68, 44, and 26, for total, evaluating, and boarding indicators, respectively.

### 3.1. Comparison between RV and Non-RV

Patient characteristics of index visits according to the RV occurrence are compared in Table 1. There were more men (46.2% vs. 43.2%, *p* = 0.0004) and older patients (*p* < 0.0001) in the RV group. The proportion of patients with KTAS 3 and 4 was higher in the RV group, whereas the proportion of KTAS 5 was higher in the non-RV group (*p* < 0.0001). The proportion of non-medical problems in the RV group was 9.7%, which was significantly lower than the 22.4% in the non-RV group (*p* < 0.0001). More laboratory study, X-rays, CT scans, and specialty consultations were performed in the RV group, and the median ED LOS was 224.5 (139.2–368.1) min in the RV group, which was longer than the 184.6 (116.5–305.2) min in the non-RV group (*p* < 0.0001). For crowding indicators of total patients, TF1h (24.7% vs. 26.4, *p* = 0.0198) and TL1h (24.0% vs. 26.2%, *p* = 0.0030) were fewer in the RV group, and TF4h did not show significant difference. All three indicators of evaluating patients were higher in the non-RV group: EF4h (30.5% vs. 32.6, *p* = 0.0060), EF1h (30.0% vs. 32.3%, *p* = 0.0147), and EL1h (29.4% vs. 31.5, *p* = 0.0076). For boarding indicators, more BF1h were in the RV group than non-RV group (20.8% vs. 19.4, *p* = 0.0354). There was no significant difference in BF4h and BL1h between the two groups.

### 3.2. Influence of ED Overcrowding on the RV Occurrence

**Figure 4:**
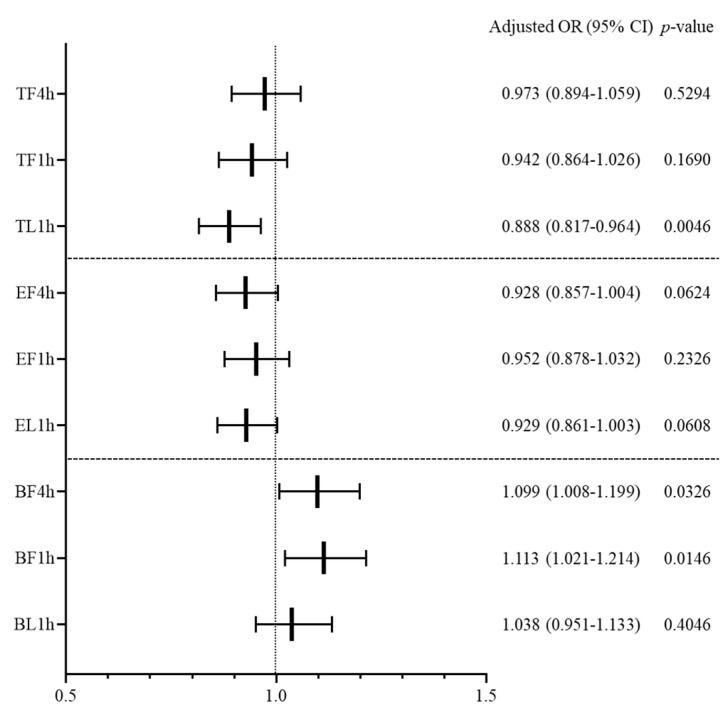
Influence of overcrowding in the emergency department on the occurrence of return visit within 72 h. OR, odds ratio; CI, confidence interval; TF4h, total patients during first 4 h; TF1h, total patients during first 1 h; TL1h, total patients during last 1 h; EF4h, evaluating patients during first 4 h; EF1h, evaluating patients during first 1 h; EL1h, evaluating patients during last 1 h; BF4h, boarding patients during first 4 h; BF1h, boarding patients during first 1 h; BL1h, boarding patients during last 1 h.

Multivariable logistic regression was conducted for each crowding indicator (Figure 4). Both TF4h and TF1h did not show a significant relation to RV occurrence, although TL1h was found to have a significant effect in decreasing RV occurrence (adjusted OR (95% CI), 0.888 (0.817–0.964), *p* = 0.0046). In the indicators of evaluating patients, the effect of overcrowding on RV was not statistically significant. Both BF4h and BF1h were found to increase RV occurrence: adjusted OR (95% CI), 1.099 (1.008–1.199), *p* = 0.0326 for BF4h; 1.113 (1.021–1.214), *p* = 0.0146 for BF1h. However, BL1h had no significant effect on RV occurrence. Other factors influencing the RV occurrence were age, gender, EMS, non-medical problem, complaint category, time of ED arrival, MRI evaluation, specialty consultation, and discharge against medical advice (DAMA). The result of univariable logistic regression for RV occurrence is presented in Appendix A, and the results of multivariable logistic regression of each crowding indicator are shown in Appendix B, Appendix C and Appendix D.

### 3.3. Comparison between RVA and RVNA

Table 2 compares the characteristics of patients with RVA and RVNA. In the RVA group, patients were older and there were more males (51.4% vs. 44.0%, *p* < 0.0001). There were more KTAS 2 and 3 in the RVA group, and more KTAS 4 and 5 in the RVNA group (*p* < 0.0001). In the RVA group, the proportion of non-medical problems was lower (4.6% vs. 11.9%, *p* < 0.0001), and more patients were diagnosed with severe disease (15.2% vs. 6.3%, *p* < 0.0001). Laboratory tests, imaging studies, and specialty consultations were carried out more frequently in the RVA group than in the RVNA group, and the ED LOS was longer in the RVA group (adjusted OR (95% CI) 324.3 (196.2–479.0) min vs. 191.5 (123.4–314.1) min, *p* < 0.0001). There was no significant difference between the two groups for crowding indicators related to total number of patients. In the evaluation indicators, EF4h and EL1h showed no significant difference, but EF1h was higher in RVA than in the RVNA group (33.7% vs. 28.9%, *p* = 0.0038).

### 3.4. Influence of ED Overcrowding on RVA Occurrence

**Figure 5:**
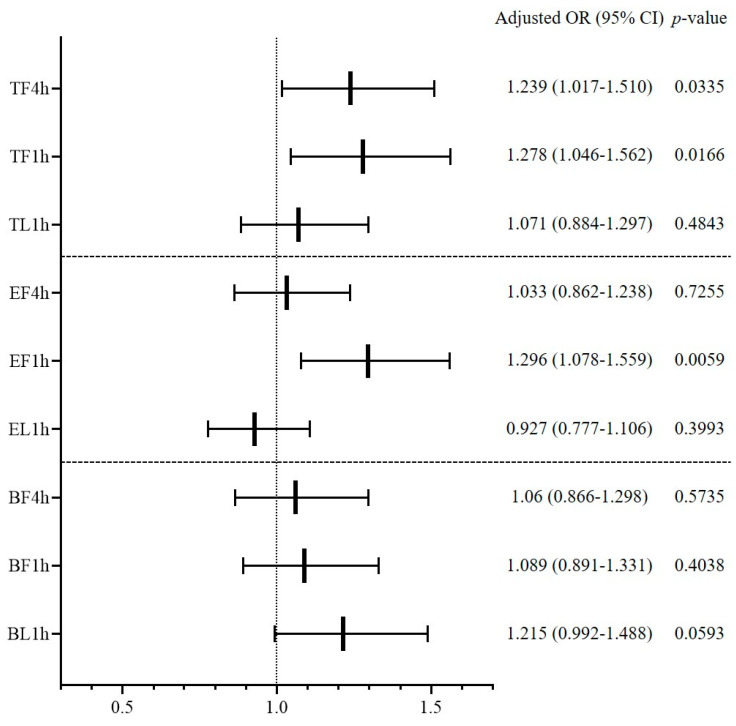
Influence of overcrowding in the emergency department on the occurrence of return visit with admission within 72 h. OR, odds ratio; CI, confidence interval; TF4h, total patients during first 4 h; TF1h, total patients during first 1 h; TL1h, total patients during last 1 h; EF4h, evaluating patients during first 4 h; EF1h, evaluating patients during first 1 h; EL1h, evaluating patients during last 1 h; BF4h, boarding patients during first 4 h; BF1h, boarding patients during first 1 h; BL1h, boarding patients during last 1 h.

Multivariable logistic regression was conducted to determine whether overcrowding indicators affected RVA occurrence (Figure 5). Among the crowding indicators of total patients, TF4h and TF1h were found to increase RVA incidence (adjusted OR (95% CI) 1.239 (1.017–1.510), *p* = 0.0335 for TF4h; 1.278 (1.046–1.562), *p* = 0.0166 for TF1h). Among evaluating indicators, EF1h was found to have a significant effect on RVA (adjusted OR (95% CI) 1.296 (1.078–1.559), *p* = 0.0059). None of the crowding indicators with regard to boarding patients had a significant effect on RVA. Other factors influencing RVA occurrence were age, sex, non-medical problem, complaint category, severe disease, treatment area, laboratory study, CT evaluation, specialty consultation, DAMA, and ED LOS. The univariable logistic regression results are presented in Appendix E, and the results of multivariable logistic regression of each crowding indicator are added as Appendix F, Appendix G and Appendix H.

## 4. Discussion

In this study, 4.3% of discharged patients returned to the ED within 72 h, which is within 2.7–6.5% of the 72 h RV rate that was previously reported [8,23,24,25]. Being male and an older adult, as well as having visits due to medical problems, were factors that increased RV, and among complaint categories, the gastrointestinal category was also a significant factor for increased RV. Previous studies examining the characteristics of RV patients have commonly reported abdominal pain as the most common cause of RV [23,24,26,27]. The factor of ED overcrowding that increased the occurrence of RV was the number of boarding patients rather than the total number of patients. Boarding patients exceeding the third quartile was associated with a 10% higher risk of RV. Accumulation of boarding patients in the ED implies that the ED output is blocked, which is directly affected by the in-hospital bed occupancy [28,29]. If the inpatient occupancy rate is too high and the ED is overcrowded with boarding patients awaiting hospital beds, emergency doctors become more careful when making a decision for additional admissions and decide to transfer some patients to other hospitals or plan discharge and short-term follow-up for patients with relatively mild conditions [30,31,32]. Previous studies have reported that high in-hospital bed occupancy reduced the number of patients admitted through the ED [31,33]. Therefore, this reduced scope of admission would probably increase the possibility of RV by increasing the risk of inadequate or premature discharge. However, one study that investigated whether inpatient occupancy affected the 72 h RV rate of ED reported results that were not significant [11]. The hospital of Sweden where the abovementioned study was conducted has a strategy to facilitate the output of the ED; the Swedish government restricts the time spent in the emergency room to 4 h, and the hospital operates a full-capacity protocol when the inpatient bed-occupancy rate reaches 100% [11]. The government’s policy to limit the ED LOS brings about changes in the hospital system that facilitates patient flow [34], and the full-capacity protocol that hospitalizes boarding patients of the ED into the corridor of hospital wards in overcrowded situation is effective in resolving ED overcrowding [35,36]. However, the Korean government is not adopting this potent policy, and most Korean hospitals, including ours, do not have effective hospital-wide action plans against overcrowding; therefore, medical staff in the ED must struggle with the burden of hospital overcrowding at their own individual strengths. We found that the number of boarding patients increased the occurrence of RV, reflecting greater pressure of the ED output blockage on emergency physicians in our ED. Operating an aftercare service for a high-risk patients discharged home when the ED output is blocked may help reduce RV.

An interesting and unexpected result in this study was that excessive numbers of total patients at the time of ED departure of index visit reduced RV occurrence. However, this reverse correlation between ED overcrowding and RV can be found in the results of McCusker et al.’s study [37]. The patient’s discharge process in the ED is an important step in explaining the patient’s condition and examination results, giving the patient confidence in the doctor’s discharge decision, and educating post-discharge precautions [38,39,40]. However, ED overcrowding shortens the time for the doctor to communicate with the patient; doctors may find it difficult to invest sufficient time to explain discharge instructions to the patient in situations where more patients need to be cared for [38,41]. If the discharge process progresses hastily, the patient may not adequately understand post-discharge instructions, which may lead to increased RV, but may also make the patient less likely to come to the ED again by reducing patient satisfaction [42,43]. This is consistent with the perspective that ED visitors are potential consumers of the hospital in the marketing aspect of medical services, and satisfaction in the ED affects the perception of the quality of medical services of the hospital, thereby improving customer loyalty and continuing to use their services [44,45]. A study analyzing the State Emergency Department Databases (SEDD) of six states in the United States reported that 32% of RV patients returned to the ED of another hospital rather than the one at the first visit [6]. The patient’s deterioration in satisfaction due to ED overcrowding can be a factor in choosing other hospitals when additional medical treatment is needed—that is, reducing the RV to our ED. It is necessary to further conduct regional multicenter research on patients who choose other hospitals when they return to the ED.

The entire 72 h RV rate has been most commonly used for monitoring ED performance [4,5,21,23], but many of the RVs are caused by patient factors such as worry about health, or illness factors such as disease progression, rather than medical error [1,9,20,23], and several researchers suggested that the rate of RVA may be a better indicator for reflecting the quality of healthcare [3,46]. In this study, the 72 h RVA rate was 0.9% of total ED visits, similar to the 0.5–1.5% reported in previous studies [1,11,20]. The proportion of RVA in total RV was 29.7%, which was higher than the admission rate of 24.0% in all ED patients. In the previous study, the admission rate of patients who returned to the ED within 72 h has been variously reported as 8.2–46.1% [3,5,19,20]. A common risk factor for RVA in previous studies was being aged ≥ 65 years, which was also confirmed in our study [8,20]. ED overcrowding, especially at the arrival time of index visit, was a significant factor causing RVA rather than RVNA. A study focusing on the adverse outcome of ED overcrowding also noted a small but significant increase in RVA as the ED occupancy increased [37]. However, two studies on the subject of disposition after RV did not confirm the significant effect of crowding on admission, which may be due to differences from our study in the definition of overcrowding [8,20]. Hu et al. used the ED census during the shift of the index visit [8], and Hayward et al. applied the ED occupancy level at the triage of the index visit as a continuous variable [20]. In McCarthy et al.’s study on the method of measuring ED crowding, crowding measured at the daily level masked most of the crowding variations that occurred within 24 h, and ED census on arrival revealed more variations in crowding compared to the average ED census during the shift [47]. We were able to confirm that the ED overcrowding is a factor that increases the occurrence of RVA by setting an influential indicator of excessive number of patients over the critical level at the time of ED arrival of the index visit. When the level of overcrowding exceeds the critical threshold, additional manpower and resources should be provided to ensure patient safety. Additionally, a system for prompt and accurate decision-making, such as early physician assessment and physician-led triage, will help prevent RV as well as alleviate ED crowding through improved patient flow [48].

### Limitation

This study had some limitations. First, as this study was conducted in a single hospital, patients who returned to other hospitals after discharge from our ED were not included. Actual revisit rates could be higher than detected, and patients who chose other hospitals may have different characteristics, which may have caused selection bias. Second, this ED belongs to a tertiary university hospital, which is located in the capital city of South Korea and has a high bed-occupancy rate and large number of patients waiting for admission. Therefore, care is warranted when seeking to generalize our results to other hospitals or countries. Third, we defined overcrowding simply on the basis of the number of occupying patients, and the difficulty of treatment or the severity of the disease of occupying patients was not taken into account. Even the same number of patients may differ in regards to burden on medical staff depending on the characteristics of the patient. Therefore, further research is needed to apply overcrowding criteria that can more accurately judge the actual overload of medical staff. Lastly, as a drawback of the retrospective study design, we could not investigate the cause of RV. In order to understand why ED overcrowding affects RV, it is necessary to look into the effect of overcrowding by the category of the cause of RV.

## 5. Conclusions

The occurrence of RV depends on ED overcrowding defined by the number of boarding patients above the third quartile. A higher number of patients occupying the ED above the third quartile at the arrival time of the index visit appeared to increase the likelihood of admission when the patient returned to the ED. However, excessive patient load when leaving the ED was associated with reduced incidence of RV. In order to understand the mechanism of this phenomenon, it would be helpful to analyze the reasons of RV together.

## Figures and Tables

**Table 1:** Comparison of patient characteristics of index visits according to the occurrence of return visit within 72 h.

Variables		RV *n* = 3743	Non-RV *n* = 83,617	*p*-Value
Age, *n* (%)	–39	1087 (29.0)	32,991 (39.5)	<0.0001
	40–64	1415 (37.8)	29,508 (35.3)	
	65–79	895 (23.9)	15,999 (19.1)	
	80–	346 (9.2)	5119 (6.1)	
Female, *n* (%)		2015 (53.8)	47,476 (56.8)	0.0004
Transfer in, *n* (%)		320 (8.5)	6818 (8.2)	0.3875
EMS, *n* (%)		858 (22.9)	15,781 (18.9)	<0.0001
KTAS, *n* (%)	1	6 (0.2)	229 (0.3)	<0.0001
	2	207 (5.5)	4566 (5.5)	
	3	797 (21.3)	15,801 (18.9)	
	4	2277 (60.8)	48,931 (58.5)	
	5	456 (12.2)	14,090 (16.9)	
Non-medical, *n* (%)		363 (9.7)	18,723 (22.4)	<0.0001
Complaint category, *n* (%)	Gastrointestinal	813 (21.7)	14,498 (17.3)	<0.0001
	General	702 (18.8)	12,440 (14.9)	
	Neurological	472 (12.6)	12,454 (14.9)	
	Musculoskeletal	289 (7.7)	8489 (10.2)	
	Cardiovascular	320 (8.5)	8068 (9.6)	
	Skin	212 (5.7)	7633 (9.1)	
	ENT	265 (7.1)	6698 (8.0)	
	Respiratory	241 (6.4)	4312 (5.2)	
	Others	429 (11.5)	9025 (10.8)	
Severe disease, *n* (%)		336 (9.0)	5946 (7.1)	<0.0001
Emergency physician, *n* (%)		1373 (36.7)	29,129 (34.8)	0.0205
Area, *n* (%)	Monitoring area	156 (4.2)	3101 (3.7)	<0.0001
	Bed area	722 (19.3)	11,158 (13.3)	
	Chair area	1016 (27.1)	19,129 (22.9)	
	Fast track	1849 (49.4)	50,229 (60.1)	
Time of ED arrival, *n* (%)	0–6	758 (20.3)	16,328 (19.5)	0.0208
	6–12	1042 (27.8)	21,896 (26.2)	
	12–18	963 (25.7)	23,117 (27.6)	
	18–24	980 (26.2)	22,276 (26.6)	
Weekend, *n* (%)		1297 (34.7)	28,871 (34.5)	0.8763
Season, *n* (%)	Spring	1093 (29.2)	23,931 (28.6)	0.0752
	Summer	759 (20.3)	15,803 (18.9)	
	Autumn	843 (22.5)	19,299 (23.1)	
	Winter	1048 (28.0)	24,584 (29.4)	
Laboratory study, *n* (%)		2877 (76.9)	55,042 (65.8)	<0.0001
Imaging study, *n* (%)	X-ray	2760 (73.7)	56,540 (67.6)	<0.0001
	CT	1163 (31.1)	24,500 (29.3)	0.0199
	MRI	93 (2.5)	4095 (4.9)	<0.0001
Specialty consultation, *n* (%)		2019 (53.9)	38,453 (46.0)	<0.0001
Discharge type, *n* (%)	Normal discharge	3415 (91.2)	79,385 (94.9)	<0.0001
	DAMA	328 (8.8)	4232 (5.1)	
ED arrival to area (min), median (IQR)		13.1 (7.9–21.0)	13.7 (8.4–21.7)	<0.0001
ED LOS (min), median (IQR)		224.5 (139.2–368.1)	184.6 (116.5–305.2)	<0.0001
Overcrowding, *n* (%)	TF4h	946 (25.3)	22,007 (26.3)	0.1553
	TF1h	924 (24.7)	22,075 (26.4)	0.0198
	TL1h	899 (24.0)	21,901 (26.2)	0.0030
	EF4h	1140 (30.5)	27,265 (32.6)	0.0060
	EF1h	1136 (30.3)	26,970 (32.3)	0.0147
	EL1h	1101 (29.4)	26,327 (31.5)	0.0076
	BF4h	757 (20.2)	15,856 (19.0)	0.0543
	BF1h	777 (20.8)	16,195 (19.4)	0.0354
	BL1h	729 (19.5)	15,804 (18.9)	0.3789

RV, return visit; EMS, emergency medical services; KTAS, Korean Triage and Acuity Scale; ENT, ears, nose and throat; ED, emergency department; CT, computed tomography; MRI, magnetic resonance imaging; DAMA, discharge against medical advice; IQR, interquartile range; LOS, length of stay; TF4h, total patients during first 4 h; TF1h, total patients during first 1 h; TL1h, total patients during last 1 h; EF4h, evaluating patients during first 4 h; EF1h, evaluating patients during first 1 h; EL1h, evaluating patients during last 1 h; BF4h, boarding patients during first 4 h; BF1h, boarding patients during first 1 h; BL1h, boarding patients during last 1 h.

**Table 2:** Comparison of patient characteristics of index visits according to the treatment result of return visit.

Variables		RVA *n* = 1113	RVNA *n* = 2630	*p*-Value
Age, *n* (%)	–39	240 (21.6)	847 (32.2)	<0.0001
	40–64	414 (37.2)	1001 (38.1)	
	65–79	333 (29.9)	562 (21.4)	
	80–	126 (11.3)	220 (8.4)	
Female, *n* (%)		541 (48.6)	1474 (56.0)	<0.0001
Transfer in, *n* (%)		117 (10.5)	203 (7.7)	0.0052
EMS, *n* (%)		298 (26.8)	560 (21.3)	0.0003
KTAS, *n* (%)	1	2 (0.2)	4 (0.2)	<0.0001
	2	76 (6.8)	131 (5.0)	
	3	324 (29.1)	473 (18.0)	
	4	655 (58.8)	1622 (61.7)	
	5	56 (5.0)	400 (15.2)	
Non-medical, *n* (%)		51 (4.6)	312 (11.9)	<0.0001
Complaint category, *n* (%)	Gastrointestinal	324 (29.1)	489 (18.6)	<0.0001
	General	248 (22.3)	454 (17.3)	
	Neurological	131 (11.8)	341 (13.0)	
	Cardiovascular	83 (7.5)	237 (9.0)	
	Musculoskeletal	61 (5.5)	228 (8.7)	
	ENT	47 (4.2)	218 (8.3)	
	Respiratory	105 (9.4)	136 (5.2)	
	Skin	15 (1.3)	197 (7.5)	
	Others	99 (8.9)	330 (12.5)	
Severe disease, *n* (%)		169 (15.2)	167 (6.3)	<0.0001
Emergency physician, *n* (%)		474 (42.6)	899 (34.2)	<0.0001
Area, *n* (%)	Monitoring area	71 (6.4)	85 (3.2)	<0.0001
	Bed area	312 (28.0)	410 (15.6)	
	Chair area	334 (30.0)	682 (25.9)	
	Fast track	396 (35.6)	1453 (55.2)	
Time of ED arrival, *n* (%)	0–6	196 (17.6)	562 (21.4)	0.0384
	6–12	331 (29.7)	711 (27.0)	
	12–18	299 (26.9)	664 (25.2)	
	18–24	287 (25.8)	693 (26.3)	
Weekend, *n* (%)		396 (35.6)	901 (34.3)	0.4376
Season, *n* (%)	Spring	323 (29.0)	770 (29.3)	0.9808
	Summer	228 (20.5)	531 (20.2)	
	Autumn	247 (22.2)	596 (22.7)	
	Winter	315 (28.3)	733 (27.9)	
Laboratory study, *n* (%)		1002 (90.0)	1875 (71.3)	<0.0001
Imaging study, *n* (%)	X-ray	958 (86.1)	1802 (68.5)	<0.0001
	CT	447 (40.2)	716 (27.2)	<0.0001
	MRI	37 (3.3)	56 (2.1)	0.0318
Specialty consultation, *n* (%)		784 (70.4)	1235 (47.0)	<0.0001
Discharge type, *n* (%)	Normal discharge	980 (88.1)	2435 (92.6)	<0.0001
	DAMA	133 (11.9)	195 (7.4)	
ED arrival to area (min), median (IQR)		13.2 (7.5–21.7)	13.1 (8.1–20.6)	0.9295
ED LOS (min), median (IQR)		324.3 (196.2–479.0)	191.5 (123.4–314.1)	<0.0001
Overcrowding, *n* (%)	TF4h	304 (27.3)	642 (24.4)	0.0618
	TF1h	298 (26.8)	626 (23.8)	0.0539
	TL1h	270 (24.3)	629 (23.9)	0.8226
	EF4h	358 (32.2)	782 (29.7)	0.1396
	EF1h	375 (33.7)	761 (28.9)	0.0038
	EL1h	324 (29.1)	777 (29.5)	0.7903
	BF4h	225 (20.2)	532 (20.2)	0.9931
	BF1h	234 (21.0)	543 (20.6)	0.7944
	BL1h	226 (20.3)	503 (19.1)	0.4047

RVA, return visit with admission; RVNA, return visit with no admission; EMS, emergency medical services; KTAS, Korean Triage and Acuity Scale; ENT, ears, nose and throat; ED, emergency department; CT, computed tomography; MRI, magnetic resonance imaging; DAMA, discharge against medical advice; IQR, interquartile range; LOS, length of stay; TF4h, total patients during first 4 h; TF1h, total patients during first 1 h; TL1h, total patients during last 1 h; EF4h, evaluating patients during first 4 h; EF1h, evaluating patients during first 1 h; EL1h, evaluating patients during last 1 h; BF4h, boarding patients during first 4 h; BF1h, boarding patients during first 1 h; BL1h, boarding patients during last 1 h.

## Appendix A

**Table A1:** Univariable logistic regression of emergency department overcrowding for return visit within 72 h.

Variable.		Odds Ratio (95% CI)	*p*-Value
Age	–39	1	
	40–64	1.455 (1.343–1.578)	<0.0001
	65–79	1.698 (1.551–1.859)	<0.0001
	80–	2.051 (1.811–2.323)	<0.0001
Female		0.888 (0.831–0.948)	0.0004
Transfer in		1.053 (0.937–1.184)	0.3876
EMS		1.279 (1.182–1.383)	<0.0001
KTAS	1	1	
	2	1.730 (0.760–3.938)	0.1914
	3	1.925 (0.853–4.343)	0.1146
	4	1.776 (0.789–3.999)	0.1654
	5	1.235 (0.546–2.793)	0.6119
Non-medical		0.372 (0.334–0.415)	<0.0001
Complaint category	Gastrointestinal	1	
	General	1.006 (0.907–1.116)	0.9054
	Neurological	0.676 (0.602–0.759)	<0.0001
	Musculoskeletal	0.607 (0.529–0.696)	<0.0001
	Cardiovascular	0.707 (0.620–0.807)	<0.0001
	Skin	0.495 (0.425–0.578)	<0.0001
	ENT	0.706 (0.612–0.813)	<0.0001
	Respiratory	0.997 (0.860–1.155)	0.9648
	Others	0.848 (0.752–0.956)	0.0069
Severe disease		1.289 (1.149–1.446)	<0.0001
Emergency physician		1.084 (1.012–1.160)	0.0205
Area	Monitoring area	1	
	Bed area	1.286 (1.077–1.536)	0.0055
	Chair area	1.056 (0.888–1.255)	0.5379
	Fast track	0.732 (0.619–0.865)	0.0003
Time of ED arrival	0–6	1	
	6–12	1.025 (0.932–1.128)	0.6118
	12–18	0.897 (0.814–0.989)	0.0290
	18–24	0.948 (0.860–1.044)	0.2770
Weekend		1.005 (0.939–1.077)	0.8762
Season	Spring	1	
	Summer	1.052 (0.957–1.156)	0.2982
	Autumn	0.956 (0.872–1.048)	0.3412
	Winter	0.933 (0.856–1.018)	0.1185
Laboratory study		1.725 (1.596–1.863)	<0.0001
Imaging study	X-ray	1.344 (1.248–1.448)	<0.0001
	CT	1.088 (1.013–1.168)	0.0200
	MRI	0.495 (0.402–0.609)	<0.0001
Specialty consultation		1.376 (1.288–1.469)	<0.0001
Discharge type	DAMA	1.802 (1.602–2.026)	<0.0001
ED arrival to area		0.930 (0.832–1.040)	0.2012
ED LOS		1.018 (1.014–1.023)	<0.0001
Overcrowding	TF4h	0.947 (0.878–1.021)	0.1553
	TF1h	0.914 (0.847–0.986)	0.0199
	TL1h	0.891 (0.825–0.962)	0.0031
	EF4h	0.905 (0.843–0.972)	0.0060
	EF1h	0.915 (0.852–0.983)	0.0147
	EL1h	0.907 (0.844–0.974)	0.0076
	BF4h	1.083 (0.999–1.176)	0.0543
	BF1h	1.091 (1.006–1.182)	0.0354
	BL1h	1.038 (0.956–1.128)	0.3738

CI, confidence interval; EMS, emergency medical services; KTAS, Korean Triage and Acuity Scale; ENT, ears, nose and throat; ED, emergency department; CT, computed tomography; MRI, magnetic resonance imaging; DAMA, discharge against medical advice; LOS, length of stay; TF4h, total patients during first 4 h; TF1h, total patients during first 1 h; TL1h, total patients during last 1 h; EF4h, evaluating patients during first 4 h; EF1h, evaluating patients during first 1 h; EL1h, evaluating patients during last 1 h; BF4h, boarding patients during first 4 h; BF1h, boarding patients during first 1 h; BL1h, boarding patients during last 1 h.

## Appendix B

**Table A2:** Multivariable logistic regression of emergency department overcrowding of the number of total patients for return visit within 72 h.

Variables		TF4h		TF1h		TL1h	
		Odds Ratio (95% CI)	*p*-Value	Odds Ratio (95% CI)	*p*-Value	Odds Ratio (95% CI)	*p*-Value
Age	–39	1		1		1	
	40–64	1.332 (1.226–1.448)	<0.0001	1.333 (1.226–1.448)	<0.0001	1.334 (1.228–1.450)	<0.0001
	65–79	1.473 (1.338–1.623)	<0.0001	1.475 (1.339–1.624)	<0.0001	1.477 (1.341–1.628)	<0.0001
	80–	1.713 (1.500–1.956)	<0.0001	1.715 (1.502–1.959)	<0.0001	1.719 (1.505–1.962)	<0.0001
Female		0.910 (0.851–0.973)	0.0058	0.910 (0.851–0.973)	0.0058	0.910 (0.851–0.973)	0.0058
EMS		1.119 (1.023–1.224)	0.0142	1.119 (1.023–1.224)	0.0142	1.119 (1.023–1.224)	0.0139
Non-medical		0.338 (0.341–0.441)	<0.0001	0.387 (0.340–0.440)	<0.0001	0.387 (0.340–0.440)	<0.0001
Complaint category	Gastrointestinal	1		1		1	
	General	1.053 (0.947–1.172)	0.3375	1.053 (0.947–1.171)	0.3433	1.052 (0.946–1.170)	0.3505
	Neurological	0.797 (0.701–0.906)	0.0005	0.797 (0.701–0.906)	0.0005	0.797 (0.701–0.906)	0.0005
	Musculoskeletal	0.984 (0.847–1.143)	0.8327	0.983 (0.846–1.141)	0.8201	0.983 (0.847–1.142)	0.8243
	Cardiovascular	0.614 (0.536–0.704)	<0.0001	0.614 (0.536–0.704)	<0.0001	0.615 (0.537–0.704)	<0.0001
	Skin	0.856 (0.724–1.013)	0.0711	0.855 (0.722–1.011)	0.0674	0.853 (0.721–1.010)	0.0646
	ENT	0.954 (0.818–1.113)	0.5503	0.952 (0.816–1.111)	0.5293	0.949 (0.814–1.108)	0.5085
	Respiratory	0.831 (0.714–0.966)	0.0162	0.830 (0.714–0.966)	0.0161	0.830 (0.714–0.966)	0.0161
	Others	0.902 (0.794–1.026)	0.1163	0.901 (0.793–1.024)	0.1109	0.900 (0.791–1.023)	0.1061
Severe disease		0.906 (0.802–1.025)	0.1166	0.907 (0.802–1.025)	0.1176	0.907 (0.802–1.025)	0.1187
Emergency physician		0.987 (0.911–1.069)	0.7417	0.989 (0.913–1.071)	0.7787	0.997 (0.920–1.080)	0.9396
Area	Monitoring area	1		1		1	
	Bed area	1.198 (0.998–1.438)	0.0522	1.196 (0.996–1.436)	0.0547	1.195 (0.995–1.434)	0.0560
	Chair area	1.076 (0.892–1.298)	0.4442	1.071 (0.888–1.292)	0.4744	1.070 (0.887–1.290)	0.4818
	Fast track	0.953 (0.792–1.148)	0.6143	0.960 (0.797–1.156)	0.6657	0.968 (0.804–1.165)	0.7303
Time of ED arrival	0–6	1		1		1	
	6–12	0.910 (0.820–1.010)	0.0757	0.908 (0.819–1.007)	0.0676	0.926 (0.834–1.028)	0.1484
	12–18	0.850 (0.762–0.948)	0.0034	0.857 (0.768–0.956)	0.0056	0.856 (0.768–0.953)	0.0047
	18–24	0.959 (0.868–1.059)	0.4063	0.963 (0.872–1.064)	0.4619	0.958 (0.867–1.058)	0.3973
Laboratory study		1.091 (0.975–1.221)	0.1273	1.094 (0.977–1.224)	0.1186	1.094 (0.978–1.224)	0.1161
Imaging study	X-ray	1.037 (0.935–1.149)	0.4920	1.038 (0.936–1.151)	0.4790	1.038 (0.936–1.151)	0.4776
	CT	1.036 (0.956–1.123)	0.3897	1.036 (0.956–1.123)	0.3885	1.034 (0.954–1.121)	0.4108
	MRI	0.403 (0.322–0.504)	<0.0001	0.403 (0.322–0.504)	<0.0001	0.401 (0.320–0.502)	<0.0001
Specialty consultation		1.297 (1.204–1.398)	<0.0001	1.297 (1.204–1.398)	<0.0001	1.296 (1.203–1.396)	<0.0001
Discharge type	DAMA	1.807 (1.600–2.041)	<0.0001	1.809 (1.601–2.043)	<0.0001	1.813 (1.605–2.048)	<0.0001
ED LOS		1.002 (0.995–1.008)	0.6045	1.002 (0.995–1.008)	0.5665	1.002 (0.996–1.009)	0.4937
Overcrowding	TF4h	0.973 (0.894–1.059)	0.5294				
	TF1h			0.942 (0.864–1.026)	0.1690		
	TL1h					0.888 (0.817–0.964)	0.0046

TF4h, total patients during first 4 h; TF1h, total patients during first 1 h; TL1h, total patients during last 1 h; CI, confidence interval; EMS, emergency medical services; ENT, ears, nose and throat; ED, emergency department; CT, computed tomography; MRI, magnetic resonance imaging; DAMA, discharge against medical advice; LOS, length of stay.

## Appendix C

**Table A3:** Multivariable logistic regression of emergency department overcrowding of the number of evaluating patients for return visit within 72 h.

Variables		EF4h		EF1h		EL1h	
		Odds Ratio (95% CI)	*p*-Value	Odds Ratio (95% CI)	*p*-Value	Odds Ratio (95% CI)	*p*-Value
Age	–39	1		1		1	
	40–64	1.332 (1.225–1.447)	<0.0001	1.332 (1.225–1.447)	<0.0001	1.331 (1.225–1.446)	<0.0001
	65–79	1.474 (1.338–1.623)	<0.0001	1.472 (1.337–1.622)	<0.0001	1.473 (1.337–1.622)	<0.0001
	80-	1.711 (1.498–1.953)	<0.0001	1.711 (1.499–1.954)	<0.0001	1.711 (1.499–1.954)	<0.0001
Female		0.910 (0.852–0.973)	0.0060	0.910 (0.852–0.973)	0.0059	0.910 (0.851–0.973)	0.0059
EMS		1.118 (1.022–1.223)	0.0150	1.118 (1.022–1.223)	0.0146	1.118 (1.022–1.223)	0.0147
Non-medical		0.387 (0.341–0.441)	<0.0001	0.388 (0.341–0.441)	<0.0001	0.388 (0.341–0.441)	<0.0001
Complaint category	Gastrointestinal	1		1		1	
	General	0.615 (0.536–0.704)	<0.0001	0.614 (0.536–0.704)	<0.0001	0.615 (0.537–0.704)	<0.0001
	Neurological	0.955 (0.819–1.114)	0.5601	0.955 (0.819–1.115)	0.5605	0.956 (0.819–1.115)	0.5667
	Musculoskeletal	1.054 (0.948–1.172)	0.3320	1.054 (0.948–1.172)	0.3329	1.054 (0.948–1.172)	0.3338
	Cardiovascular	0.985 (0.848–1.144)	0.8407	0.984 (0.848–1.143)	0.8359	0.985 (0.848–1.144)	0.8457
	Skin	0.797 (0.701–0.906)	0.0005	0.797 (0.702–0.906)	0.0005	0.799 (0.703–0.908)	0.0006
	ENT	0.902 (0.794–1.026)	0.1157	0.903 (0.794–1.026)	0.1175	0.904 (0.795–1.027)	0.1215
	Respiratory	0.831 (0.714–0.967)	0.0164	0.831 (0.714–0.967)	0.0164	0.830 (0.714–0.966)	0.0160
	Others	0.857 (0.724–1.015)	0.0733	0.857 (0.724–1.014)	0.0731	0.858 (0.725–1.015)	0.0748
Severe disease		0.905 (0.801–1.024)	0.1122	0.906 (0.801–1.024)	0.1134	0.906 (0.801–1.024)	0.1132
Emergency physician		0.988 (0.912–1.069)	0.7592	0.985 (0.910–1.066)	0.7039	0.987 (0.911–1.068)	0.7425
Area	Monitoring area	1		1		1	
	Bed area	1.202 (1.001–1.442)	0.0486	1.200 (1.000–1.441)	0.0498	1.203 (1.002–1.444)	0.0474
	Chair area	1.076 (0.892–1.297)	0.4444	1.075 (0.892–1.297)	0.4473	1.078 (0.894–1.300)	0.4308
	Fast track	0.955 (0.794–1.149)	0.6276	0.952 (0.791–1.145)	0.6031	0.953 (0.792–1.146)	0.6092
Time of ED arrival	0–6	1		1		1	
	6–12	0.919 (0.828–1.020)	0.1134	0.909 (0.819–1.008)	0.0701	0.928 (0.835–1.032)	0.1684
	12–18	0.875 (0.781–0.981)	0.0218	0.864 (0.771–0.967)	0.0111	0.868 (0.777–0.970)	0.0125
	18–24	0.977 (0.883–1.082)	0.6598	0.972 (0.877–1.078)	0.5945	0.968 (0.875–1.069)	0.5184
Laboratory study		1.090 (0.975–1.220)	0.1311	1.091 (0.975–1.220)	0.1303	1.089 (0.973–1.218)	0.1375
Imaging study	X-ray	1.038 (0.936–1.150)	0.4821	1.037 (0.936–1.150)	0.4863	1.037 (0.935–1.149)	0.4902
	CT	1.036 (0.956–1.122)	0.3897	1.036 (0.956–1.122)	0.3934	1.035 (0.955–1.121)	0.4056
	MRI	0.402 (0.322–0.503)	<0.0001	0.403 (0.322–0.504)	<0.0001	0.402 (0.321–0.503)	<0.0001
Specialty consultation		1.298 (1.204–1.398)	<0.0001	1.297 (1.204–1.398)	<0.0001	1.298 (1.205–1.399)	<0.0001
Discharge type	DAMA	1.810 (1.603–2.045)	<0.0001	1.808 (1.601–2.042)	<0.0001	1.809 (1.601–2.043)	<0.0001
ED LOS		1.002 (0.995–1.008)	0.5750	1.002 (0.995–1.008)	0.5971	1.002 (0.995–1.008)	0.6427
Overcrowding	EF4h	0.928 (0.857–1.004)	0.0624				
	EF1h			0.952 (0.878–1.032)	0.2326		
	EL1h					0.929 (0.861–1.003)	0.0608

EF4h, evaluating patients during first 4 h; EF1h, evaluating patients during first 1 h; EL1h, evaluating patients during last 1 h; CI, confidence interval; EMS, emergency medical services; ENT, ears, nose and throat; ED, emergency department; CT, computed tomography; MRI, magnetic resonance imaging; DAMA, discharge against medical advice; LOS, length of stay.

## Appendix D

**Table A4:** Multivariable logistic regression of emergency department overcrowding of the number of boarding patients for return visit within 72 h.

Variables		BF4h		BF1h		BL1h	
		Odds Ratio (95% CI)	*p*-Value	Odds Ratio (95% CI)	*p*-Value	Odds Ratio (95% CI)	*p*-Value
Age	–39	1		1		1	
	40–64	1.330 (1.224–1.445)	<0.0001	1.329 (1.223–1.445)	<0.0001	1.331 (1.224–1.446)	<0.0001
	65–79	1.468 (1.333–1.617)	<0.0001	1.467 (1.332–1.616)	<0.0001	1.471 (1.335–1.620)	<0.0001
	80-	1.703 (1.491–1.945)	<0.0001	1.702 (1.490–1.943)	<0.0001	1.708 (1.496–1.951)	<0.0001
Female		0.910 (0.851–0.973)	0.0058	0.910 (0.851–0.973)	0.0057	0.910 (0.851–0.973)	0.0058
EMS		1.117 (1.022–1.222)	0.0151	1.117 (1.021–1.222)	0.0153	1.118 (1.022–1.223)	0.0145
Non-medical		0.390 (0.342–0.443)	<0.0001	0.390 (0.343–0.443)	<0.0001	0.389 (0.342–0.442)	<0.0001
Complaint category	Gastrointestinal	1		1		1	
	General	1.055 (0.949–1.173)	0.3240	1.055 (0.949–1.174)	0.3217	1.055 (0.948–1.173)	0.3272
	Neurological	0.799 (0.703–0.908)	0.0006	0.799 (0.703–0.908)	0.0006	0.798 (0.702–0.907)	0.0006
	Musculoskeletal	0.987 (0.850–1.146)	0.8611	0.987 (0.850–1.147)	0.8667	0.986 (0.849–1.145)	0.8505
	Cardiovascular	0.614 (0.536–0.703)	<0.0001	0.614 (0.536–0.703)	<0.0001	0.614 (0.536–0.704)	<0.0001
	Skin	0.862 (0.728–1.020)	0.0830	0.863 (0.729–1.021)	0.0853	0.859 (0.726–1.017)	0.0777
	ENT	0.962 (0.824–1.122)	0.6219	0.963 (0.825–1.124)	0.6313	0.958 (0.821–1.118)	0.5892
	Respiratory	0.830 (0.713–0.965)	0.0157	0.830 (0.713–0.966)	0.0159	0.830 (0.714–0.966)	0.0161
	Others	0.907 (0.798–1.031)	0.1356	0.908 (0.798–1.032)	0.1381	0.905 (0.796–1.029)	0.1264
Severe disease		0.905 (0.800–1.023)	0.1091	0.905 (0.800–1.023)	0.1088	0.906 (0.801–1.024)	0.1138
Emergency physician		0.974 (0.900–1.055)	0.5246	0.973 (0.898–1.054)	0.5006	0.980 (0.905–1.062)	0.6230
Area	Monitoring area	1		1		1	
	Bed area	1.210 (1.008–1.452)	0.0408	1.212 (1.009–1.454)	0.0394	1.204 (1.003–1.446)	0.0461
	Chair area	1.090 (0.903–1.314)	0.3691	1.092 (0.905–1.317)	0.3584	1.084 (0.898–1.307)	0.4011
	Fast track	0.933 (0.776–1.123)	0.4653	0.931 (0.774–1.121)	0.4502	0.943 (0.784–1.135)	0.5336
Time of ED arrival	0–6	1		1		1	
	6–12	0.909 (0.819–1.008)	0.0709	0.908 (0.819–1.008)	0.0694	0.910 (0.820–1.009)	0.0732
	12–18	0.856 (0.768–0.953)	0.0047	0.853 (0.766–0.950)	0.0038	0.850 (0.763–0.947)	0.0033
	18–24	0.968 (0.876–1.070)	0.5201	0.968 (0.876–1.070)	0.5258	0.961 (0.869–1.062)	0.4318
Laboratory study		1.085 (0.969–1.214)	0.1562	1.083 (0.968–1.212)	0.1628	1.088 (0.972–1.217)	0.1411
Imaging study	X-ray	1.034 (0.933–1.146)	0.5269	1.034 (0.932–1.146)	0.5297	1.035 (0.934–1.148)	0.5094
	CT	1.035 (0.955–1.121)	0.4063	1.034 (0.954–1.121)	0.4107	1.036 (0.956–1.122)	0.3908
	MRI	0.403 (0.322–0.505)	<0.0001	0.403 (0.322–0.505)	<0.0001	0.403 (0.322–0.505)	<0.0001
Specialty consultation		1.298 (1.205–1.399)	<0.0001	1.298 (1.205–1.399)	<0.0001	1.298 (1.205–1.399)	<0.0001
Discharge type	DAMA	1.803 (1.596–2.037)	<0.0001	1.803 (1.596–2.037)	<0.0001	1.805 (1.598–2.039)	<0.0001
ED LOS		1.001 (0.995–1.008)	0.6975	1.001 (0.995–1.008)	0.7010	1.001 (0.995–1.008)	0.6944
Overcrowding	BF4h	1.099 (1.008–1.199)	0.0326				
	BF1h			1.113 (1.021–1.214)	0.0146		
	BL1h					1.038 (0.951–1.133)	0.4046

BF4h, boarding patients during first 4 h; BF1h, boarding patients during first 1 h; BL1h, boarding patients during last 1 h; CI, confidence interval; EMS, emergency medical services; ENT, ears, nose and throat; ED, emergency department; CT, computed tomography; MRI, magnetic resonance imaging; DAMA, discharge against medical advice; LOS, length of stay.

## Appendix E

**Table A5:** Univariable logistic regression of emergency department overcrowding for return visit with admission.

Variables		Odds Ratio (95% CI)	*p*-Value
Age	–39	1	
	40–64	1.459 (1.215–1.753)	<0.0001
	65–79	2.091 (1.716–2.546)	<0.0001
	80-	2.021 (1.555–2.625)	<0.0001
Female		0.742 (0.645–0.854)	<0.0001
Transfer in		1.404 (1.106–1.784)	0.0054
EMS		1.352 (1.149–1.590)	0.0003
KTAS	1	1	
	2	1.160 (0.208–6.485)	0.8655
	3	1.370 (0.249–7.524)	0.7172
	4	0.808 (0.148–4.420)	0.8054
	5	0.280 (0.050–1.564)	0.1470
Non-medical		0.357 (0.263–0.484)	<0.0001
Complaint category	Gastrointestinal	1	
	General	0.529 (0.397–0.704)	<0.0001
	Neurological	0.325 (0.230–0.459)	<0.0001
	Cardiovascular	0.824 (0.669–1.016)	0.0702
	Musculoskeletal	0.404 (0.295–0.554)	<0.0001
	ENT	0.580 (0.454–0.741)	<0.0001
	Respiratory	0.115 (0.067–0.198)	<0.0001
	Skin	0.453 (0.347–0.59)	<0.0001
	Others	1.165 (0.871–1.558)	0.3026
Severe disease		2.640 (2.104–3.311)	<0.0001
Emergency physician		1.428 (1.237–1.649)	<0.0001
Area	Monitoring area	1	
	Bed area	0.911 (0.643–1.290)	0.5995
	Chair area	0.586 (0.417–0.825)	0.0022
	Fast track	0.326 (0.234–0.456)	<0.0001
Time of ED arrival	0–6	1	
	6–12	1.335 (1.084–1.644)	0.0066
	12–18	1.291 (1.044–1.597)	0.0183
	18–24	1.187 (0.960–1.469)	0.1138
Weekend		1.060 (0.915–1.227)	0.4376
Season	Spring	1	
	Summer	1.024 (0.836–1.253)	0.8211
	Autumn	0.988 (0.811–1.203)	0.9047
	Winter	1.024 (0.851–1.233)	0.7979
Laboratory study		3.634 (2.936–4.499)	<0.0001
Imaging study	X-ray	2.840 (2.352–3.429)	<0.0001
	CT	1.794 (1.548–2.079)	<0.0001
	MRI	1.581 (1.037–2.409)	0.0331
Specialty consultation		2.692 (2.317–3.127)	<0.0001
Discharge type	DAMA	1.695 (1.343–2.139)	<0.0001
ED arrival to area		1.126 (0.924–1.373)	0.2400
ED LOS		1.127 (1.106–1.149)	<0.0001
Overcrowding	TF4h	1.164 (0.992–1.364)	0.0619
	TF1h	1.171 (0.997–1.374)	0.0540
	TL1h	1.019 (0.865–1.200)	0.8223
	EF4h	1.121 (0.963–1.303)	0.1396
	EF1h	1.248 (1.074–1.450)	0.0038
	EL1h	0.979 (0.839–1.142)	0.7907
	BF4h	0.999 (0.839–1.190)	0.9931
	BF1h	1.023 (0.861–1.216)	0.7939
	BL1h	1.077 (0.904–1.284)	0.4048

CI, confidence interval; EMS, emergency medical services; KTAS, Korean Triage and Acuity Scale; ENT, ears, nose and throat; ED, emergency department; CT, computed tomography; MRI, magnetic resonance imaging; DAMA, discharge against medical advice; LOS, length of stay; TF4h, total patients during first 4 h; TF1h, total patients during first 1 h; TL1h, total patients during last 1 h; EF4h, evaluating patients during first 4 h; EF1h, evaluating patients during first 1 h; EL1h, evaluating patients during last 1 h; BF4h, boarding patients during first 4 h; BF1h, boarding patients during first 1 h; BL1h, boarding patients during last 1 h.

## Appendix F

**Table A6:** Multivariable logistic regression of emergency department overcrowding of the number of total patients for return visit with admission.

Variables		TF4h		TF1h		TL1h	
		Odds Ratio (95% CI)	*p*-Value	Odds Ratio (95% CI)	*p*-Value	Odds Ratio (95% CI)	*p*-Value
Age	–39	1		1		1	
	40–64	1.094 (0.895–1.336)	0.3815	1.091 (0.893–1.333)	0.3945	1.094 (0.896–1.337)	0.3787
	65–79	1.438 (1.155–1.791)	0.0012	1.436 (1.153–1.788)	0.0012	1.441 (1.158–1.794)	0.0011
	80-	1.287 (0.961–1.725)	0.0909	1.281 (0.956–1.717)	0.0968	1.305 (0.975–1.748)	0.0736
Female		0.782 (0.671–0.912)	0.0017	0.784 (0.673–0.914)	0.0019	0.781 (0.67–0.911)	0.0016
Transfer in		0.981 (0.754–1.277)	0.8891	0.983 (0.756–1.279)	0.8988	0.995 (0.765–1.294)	0.9718
EMS		0.949 (0.779–1.158)	0.6075	0.950 (0.779–1.158)	0.6124	0.952 (0.781–1.161)	0.6270
Non-medical		0.522 (0.367–0.744)	0.0003	0.518 (0.363–0.738)	0.0003	0.520 (0.365–0.585)	0.0003
Complaint category	Gastrointestinal	1		1		1	
	General	0.840 (0.669–1.053)	0.1302	0.844 (0.673–1.058)	0.1419	0.833 (0.664–1.045)	0.1135
	Neurological	0.501 (0.375–0.669)	<0.0001	0.500 (0.374–0.667)	<0.0001	0.494 (0.37–0.66)	<0.0001
	Cardiovascular	0.432 (0.317–0.588)	<0.0001	0.433 (0.318–0.590)	<0.0001	0.429 (0.315–0.585)	<0.0001
	Musculoskeletal	0.662 (0.462–0.951)	0.0254	0.665 (0.463–0.954)	0.0268	0.659 (0.459–0.946)	0.0236
	ENT	0.451 (0.307–0.663)	<0.0001	0.456 (0.311–0.670)	<0.0001	0.447 (0.305–0.656)	<0.0001
	Respiratory	0.766 (0.557–1.055)	0.1026	0.765 (0.556–1.053)	0.1007	0.765 (0.556–1.053)	0.1007
	Skin	0.203 (0.115–0.361)	<0.0001	0.206 (0.116–0.366)	<0.0001	0.201 (0.114–0.357)	<0.0001
	Others	0.456 (0.340–0.611)	<0.0001	0.457 (0.341–0.613)	<0.0001	0.452 (0.338–0.606)	<0.0001
Severe disease		1.317 (1.020–1.702)	0.0348	1.318 (1.020–1.703)	0.0344	1.32 (1.022–1.705)	0.0331
Emergency physician		0.932 (0.778–1.117)	0.4465	0.941 (0.786–1.126)	0.5041	0.951 (0.794–1.14)	0.5896
Area	Monitoring area	1		1		1	
	Bed area	1.024 (0.700–1.499)	0.9008	1.031 (0.705–1.509)	0.8742	1.01 (0.691–1.478)	0.9581
	Chair area	0.840 (0.565–1.248)	0.3878	0.848 (0.571–1.261)	0.4156	0.825 (0.556–1.226)	0.3416
	Fast track	0.573 (0.386–0.850)	0.0057	0.572 (0.385–0.848)	0.0055	0.597 (0.403–0.885)	0.0101
Time of ED arrival	0–6	1		1		1	
	6–12	1.163 (0.914–1.480)	0.2183	1.183 (0.931–1.504)	0.1696	1.177 (0.925–1.497)	0.1849
	12–18	1.153 (0.892–1.492)	0.2773	1.134 (0.875–1.469)	0.3422	1.196 (0.927–1.544)	0.1682
	18–24	1.141 (0.901–1.445)	0.2734	1.123 (0.885–1.423)	0.3400	1.166 (0.921–1.476)	0.2024
Laboratory study		1.483 (1.113–1.976)	0.0071	1.470 (1.103–1.959)	0.0085	1.483 (1.113–1.975)	0.0071
Imaging study	X-ray	1.188 (0.921–1.532)	0.1846	1.191 (0.923–1.536)	0.1787	1.196 (0.927–1.542)	0.1683
	CT	1.279 (1.071–1.527)	0.0066	1.284 (1.075–1.534)	0.0058	1.281 (1.073–1.53)	0.0063
	MRI	0.934 (0.568–1.534)	0.7869	0.940 (0.572–1.544)	0.8056	0.941 (0.572–1.547)	0.8096
Specialty consultation		2.008 (1.679–2.400)	<0.0001	2.009 (1.680–2.401)	<0.0001	2.005 (1.677–2.398)	<0.0001
Discharge type	DAMA	1.701 (1.308–2.213)	<0.0001	1.705 (1.311–2.219)	<0.0001	1.705 (1.311–2.217)	<0.0001
ED LOS		1.023 (1.004–1.043)	0.0204	1.023 (1.003–1.043)	0.0219	1.025 (1.005–1.045)	0.0145
Overcrowding	TF4h	1.239 (1.017–1.510)	0.0335				
	TF1h			1.278 (1.046–1.562)	0.0166		
	TL1h					1.071 (0.884–1.297)	0.4843

TF4h, total patients during first 4 h; TF1h, total patients during first 1 h; TL1h, total patients during last 1 h; CI, confidence interval; EMS, emergency medical services; ENT, ears, nose and throat; ED, emergency department; CT, computed tomography; MRI, magnetic resonance imaging; DAMA, discharge against medical advice; LOS, length of stay.

## Appendix G

**Table A7:** Multivariable logistic regression of emergency department overcrowding of the number of evaluating patients for return visit with admission.

Variables		EF4h		EF1h		EL1h	
		Odds Ratio (95% CI)	*p*-Value	Odds Ratio (95% CI)	*p*-Value	Odds Ratio (95% CI)	*p*-Value
Age	–39	1		1		1	
	40–64	1.094 (0.896–1.337)	0.3770	1.098 (0.898–1.342)	0.3612	1.093 (0.895–1.336)	0.3839
	65–79	1.443 (1.159–1.797)	0.0010	1.447 (1.162–1.802)	0.0010	1.445 (1.161–1.799)	0.0010
	80-	1.307 (0.976–1.750)	0.0726	1.312 (0.979–1.758)	0.0687	1.306 (0.976–1.750)	0.0728
Female		0.780 (0.669–0.909)	0.0015	0.782 (0.670–0.911)	0.0017	0.779 (0.668–0.908)	0.0014
Transfer in		0.997 (0.767–1.297)	0.9834	1.002 (0.770–1.304)	0.9877	0.995 (0.765–1.294)	0.9713
EMS		0.951 (0.780–1.159)	0.6183	0.953 (0.782–1.163)	0.6370	0.949 (0.779–1.157)	0.6069
Non-medical		0.520 (0.365–0.741)	0.0003	0.526 (0.369–0.750)	0.0004	0.521 (0.366–0.742)	0.0003
Complaint category	Gastrointestinal						
	General	0.429 (0.315–0.584)	<0.0001	0.426 (0.313–0.581)	<0.0001	0.430 (0.315–0.586)	<0.0001
	Neurological	0.446 (0.304–0.655)	<0.0001	0.448 (0.305–0.657)	<0.0001	0.444 (0.303–0.652)	<0.0001
	Cardiovascular	0.832 (0.663–1.043)	0.1104	0.829 (0.661–1.039)	0.1041	0.833 (0.665–1.045)	0.1141
	Musculoskeletal	0.659 (0.459–0.945)	0.0233	0.663 (0.461–0.951)	0.0257	0.659 (0.460–0.946)	0.0237
	ENT	0.494 (0.370–0.660)	<0.0001	0.495 (0.371–0.662)	<0.0001	0.497 (0.372–0.664)	<0.0001
	Respiratory	0.452 (0.338–0.606)	<0.0001	0.454 (0.338–0.608)	<0.0001	0.451 (0.337–0.604)	<0.0001
	Skin	0.766 (0.556–1.054)	0.1014	0.771 (0.560–1.062)	0.1110	0.769 (0.558–1.058)	0.1063
	Others	0.201 (0.113–0.357)	<0.0001	0.199 (0.112–0.353)	<0.0001	0.201 (0.113–0.356)	<0.0001
Severe disease		1.321 (1.023–1.706)	0.0329	1.320 (1.022–1.705)	0.0335	1.323 (1.024–1.709)	0.0319
Emergency physician		0.959 (0.802–1.147)	0.6464	0.951 (0.795–1.137)	0.5792	0.964 (0.806–1.153)	0.6876
Area	Monitoring area						
	Bed area	1.005 (0.687–1.469)	0.9810	1.006 (0.688–1.471)	0.9753	1.003 (0.686–1.467)	0.9884
	Chair area	0.821 (0.553–1.219)	0.3288	0.840 (0.566–1.247)	0.3867	0.815 (0.549–1.211)	0.3120
	Fast track	0.602 (0.407–0.891)	0.0112	0.592 (0.400–0.876)	0.0087	0.605 (0.409–0.895)	0.0119
Time of ED arrival	0–6						
	6–12	1.178 (0.926–1.500)	0.1826	1.180 (0.928–1.499)	0.1772	1.211 (0.948–1.547)	0.1256
	12–18	1.182 (0.904–1.545)	0.2214	1.078 (0.825–1.406)	0.5829	1.231 (0.948–1.599)	0.1197
	18–24	1.152 (0.906–1.465)	0.2493	1.069 (0.838–1.363)	0.5929	1.170 (0.924–1.481)	0.1925
Laboratory study		1.486 (1.115–1.979)	0.0068	1.505 (1.129–2.006)	0.0054	1.490 (1.118–1.985)	0.0065
Imaging study	X-ray	1.197 (0.928–1.544)	0.1656	1.187 (0.920–1.532)	0.1862	1.194 (0.926–1.540)	0.1710
	CT	1.277 (1.069–1.525)	0.0069	1.273 (1.065–1.520)	0.0078	1.276 (1.068–1.523)	0.0072
	MRI	0.936 (0.570–1.539)	0.7954	0.938 (0.571–1.541)	0.8009	0.934 (0.569–1.536)	0.7892
Specialty consultation		2.001 (1.674–2.392)	<0.0001	2.001 (1.674–2.393)	<0.0001	1.997 (1.670–2.387)	<0.0001
Discharge type	DAMA	1.704 (1.310–2.217)	<0.0001	1.691 (1.299–2.200)	<0.0001	1.702 (1.308–2.214)	<0.0001
ED LOS		1.025 (1.005–1.045)	0.0132	1.023 (1.004–1.043)	0.0183	1.025 (1.006–1.046)	0.0113
Overcrowding	EF4h	1.033 (0.862–1.238)	0.7255				
	EF1h			1.296 (1.078–1.559)	0.0059		
	EL1h					0.927 (0.777–1.106)	0.3993

EF4h, evaluating patients during first 4 h; EF1h, evaluating patients during first 1 h; EL1h, evaluating patients during last 1 h; CI, confidence interval; EMS, emergency medical services; ENT, ears, nose and throat; ED, emergency department; CT, computed tomography; MRI, magnetic resonance imaging; DAMA, discharge against medical advice; LOS, length of stay.

## Appendix H

**Table A8:** Multivariable logistic regression of emergency department overcrowding of the number of boarding patients for return visit with admission.

Variables		BF4h		BF1h		BL1h	
		Odds Ratio (95% CI)	*p*-Value	Odds Ratio (95% CI)	*p*-Value	Odds Ratio (95% CI)	*p*-Value
Age	–39	1		1		1	
	40–64	1.094 (0.895–1.336)	0.3807	1.093 (0.895–1.336)	0.3838	1.091 (0.893–1.334)	0.3923
	65–79	1.442 (1.158–1.795)	0.0011	1.439 (1.156–1.792)	0.0011	1.439 (1.155–1.791)	0.0011
	80-	1.303 (0.973–1.745)	0.0759	1.301 (0.971–1.742)	0.0778	1.295 (0.967–1.734)	0.0827
Female		0.780 (0.669–0.909)	0.0015	0.779 (0.668–0.908)	0.0014	0.782 (0.671–0.912)	0.0017
Transfer in		0.995 (0.765–1.294)	0.9689	0.993 (0.764–1.292)	0.9588	0.989 (0.760–1.286)	0.9339
EMS		0.950 (0.779–1.158)	0.6096	0.948 (0.778–1.156)	0.6008	0.947 (0.777–1.155)	0.5920
Non-medical		0.522 (0.367–0.744)	0.0003	0.524 (0.368–0.746)	0.0003	0.527 (0.370–0.751)	0.0004
Complaint category	Gastrointestinal	1		1		1	
	General	0.834 (0.665–1.045)	0.1152	0.835 (0.666–1.047)	0.1179	0.838 (0.668–1.051)	0.1260
	Neurological	0.496 (0.371–0.662)	<0.0001	0.495 (0.371–0.662)	<0.0001	0.498 (0.373–0.665)	<0.0001
	Cardiovascular	0.430 (0.315–0.585)	<0.0001	0.429 (0.315–0.585)	<0.0001	0.433 (0.318–0.590)	<0.0001
	Musculoskeletal	0.659 (0.459–0.946)	0.0236	0.659 (0.459–0.946)	0.0236	0.662 (0.461–0.950)	0.0252
	ENT	0.447 (0.305–0.657)	<0.0001	0.448 (0.305–0.658)	<0.0001	0.451 (0.307–0.662)	<0.0001
	Respiratory	0.767 (0.557–1.055)	0.1028	0.766 (0.557–1.054)	0.1021	0.768 (0.558–1.058)	0.1061
	Skin	0.202 (0.114–0.358)	<0.0001	0.202 (0.114–0.358)	<0.0001	0.204 (0.115–0.362)	<0.0001
	Others	0.453 (0.338–0.606)	<0.0001	0.453 (0.338–0.607)	<0.0001	0.453 (0.338–0.608)	<0.0001
Severe disease		1.318 (1.020–1.702)	0.0345	1.317 (1.020–1.700)	0.0350	1.320 (1.022–1.704)	0.0334
Emergency physician		0.953 (0.795–1.142)	0.6037	0.950 (0.792–1.138)	0.5762	0.939 (0.784–1.125)	0.4970
Area	Monitoring area	1		1		1	
	Bed area	1.01 (0.690–1.477)	0.9609	1.012 (0.692–1.480)	0.9519	1.032 (0.705–1.511)	0.8713
	Chair area	0.824 (0.554–1.223)	0.3361	0.825 (0.556–1.226)	0.3419	0.840 (0.565–1.249)	0.3888
	Fast track	0.597 (0.403–0.885)	0.0103	0.593 (0.400–0.880)	0.0094	0.588 (0.397–0.872)	0.0082
Time of ED arrival	0–6	1		1		1	
	6–12	1.187 (0.934–1.510)	0.1605	1.188 (0.935–1.510)	0.1595	1.205 (0.947–1.533)	0.1287
	12–18	1.210 (0.936–1.565)	0.1447	1.210 (0.937–1.562)	0.1442	1.245 (0.962–1.611)	0.0962
	18–24	1.170 (0.923–1.483)	0.1935	1.174 (0.926–1.487)	0.1850	1.187 (0.937–1.504)	0.1565
Laboratory study		1.486 (1.116–1.979)	0.0068	1.485 (1.115–1.979)	0.0068	1.486 (1.116–1.980)	0.0068
Imaging study	X-ray	1.195 (0.926–1.541)	0.1703	1.193 (0.925–1.538)	0.1748	1.190 (0.923–1.535)	0.1794
	CT	1.278 (1.070–1.526)	0.0068	1.277 (1.069–1.525)	0.0070	1.282 (1.073–1.531)	0.0062
	MRI	0.935 (0.569–1.536)	0.7899	0.935 (0.569–1.537)	0.7907	0.951 (0.578–1.565)	0.8437
Specialty consultation		2.001 (1.674–2.393)	<0.0001	2.002 (1.675–2.394)	<0.0001	2.013 (1.684–2.408)	<0.0001
Discharge type	DAMA	1.704 (1.310–2.216)	<0.0001	1.704 (1.310–2.216)	<0.0001	1.704 (1.310–2.216)	<0.0001
ED LOS		1.025 (1.005–1.045)	0.0128	1.025 (1.005–1.045)	0.0128	1.024 (1.004–1.044)	0.0180
Overcrowding	BF4h	1.060 (0.866–1.298)	0.5735				
	BF1h			1.089 (0.891–1.331)	0.4038		
	BL1h					1.215 (0.992–1.488)	0.0593

BF4h, boarding patients during first 4 h; BF1h, boarding patients during first 1 h; BL1h, boarding patients during last 1 h; CI, confidence interval; EMS, emergency medical services; ENT, ears, nose and throat; ED, emergency department; CT, computed tomography; MRI, magnetic resonance imaging; DAMA, discharge against medical advice; LOS, length of stay.
